# Neonatal immature gastric teratoma: a case report

**DOI:** 10.3389/fped.2026.1824435

**Published:** 2026-04-29

**Authors:** Shiqi Liu, Jian Ji, Siqi Li, Yinmin Sun, Yufeng Li

**Affiliations:** 1 Medical College, Xijing University, Xi’an, Shaanxi, China; 2 Department of Neonatology, Hanzhong People’s Hospital, Hanzhong, Shaanxi, China; 3 The Second Clinical Medical School, Shaanxi University of Chinese Medicine, Xianyang, Shaanxi, China; 4 Department of Pediatric Surgery, Guilin Maternal and Child Health Hospital, Guilin, Guangxi Zhuang Autonomous Region, China

**Keywords:** gastric teratomas, immature teratoma, neonate, surgical resection, tumor markers

## Abstract

**Background:**

Gastric teratomas (GTs) are exceedingly rare neoplasms, accounting for less than 1% of all teratomas in children. Notably, GT arising from the anterior gastric wall is even rarer, with only a handful of cases reported in the literature, and the transmural growth pattern of such tumors further adds to their clinical uniqueness.

**Case presentation:**

We report a case of immature gastric teratoma arising from the anterior gastric wall in a term male neonate. The mass was detected on prenatal ultrasonography at 37 weeks and 6 days of gestation, and the infant was admitted on the first day of life for evaluation. Abdominal computed tomography revealed a heterogeneous mass measuring 51.9 mm × 47 mm × 39 mm in the hepatogastric space, containing calcifications and fatty components with close adhesion to the gastric wall; the transmural growth characteristic of the tumor was subsequently confirmed during surgical exploration. Laboratory studies demonstrated markedly elevated alpha-fetoprotein (AFP) (>60,500 ng/mL), neuron-specific enolase (NSE) (63.8 ng/mL), and lactate dehydrogenase (LDH) (575 U/L), which were suggestive of a germ cell tumor with comprehensive reference to clinical manifestations and imaging findings. The patient underwent complete surgical resection on postnatal day four. Histopathological examination confirmed a grade III immature teratoma with negative resection margins. Postoperative recovery was uneventful, with declining inflammatory markers at one-week follow-up.

**Conclusions:**

This case highlights the importance of including gastric teratoma in the differential diagnosis of neonatal abdominal masses, the diagnostic value of integrated imaging and tumor marker evaluation, and the necessity of complete surgical resection. Furthermore, it demonstrates the critical role of multidisciplinary collaboration in the timely diagnosis and management of rare neonatal tumors.

## 1 Introduction

Teratomas are tumors derived from pluripotent germ cells and represent the most common germ cell tumors in the pediatric population. They occur at a wide variety of sites, most frequently in the sacrococcygeal region, mediastinum, retroperitoneum, and central nervous system. Among these, sacrococcygeal and mediastinal teratomas are the most common, with relatively abundant clinical experience in diagnosis and treatment (1). In contrast, gastric teratoma (GT), a distinct subtype of teratoma, is extremely rare. It arises from multipotent stem cells within the gastric wall, occurs predominantly in male infants, accounting for less than 1% of all pediatric teratomas. GT can be classified as benign or malignant, with immature teratomas mostly being malignant or potentially malignant (2, 3).

We report a case of immature gastric teratoma arising from the anterior gastric wall in a term male neonate, aiming to discuss its diagnostic and therapeutic features and provide practical references for managing this rare condition.

## 2 Case report

### 2.1 Patient information

A male infant was delivered at 40 weeks and 3 days of gestation via spontaneous vaginal delivery with a birth weight of 3.3 kg. He was admitted to our institution on the first day of life for evaluation of a prenatally detected abdominal mass, initially identified on routine prenatal ultrasonography at 37 weeks and 6 days of gestation and confirmed postnatally as an intra-abdominal space-occupying lesion. The infant demonstrated no nausea, vomiting, or abdominal distension. He tolerated oral feeding well, passed meconium within 12 h of birth, and maintained normal urinary output. Both parents were healthy with no significant medical history, and there was no family history of genetic disorders or congenital anomalies. Physical examination was unremarkable apart from the imaging findings described below.

### 2.2 Imaging findings

**Figure 1:**
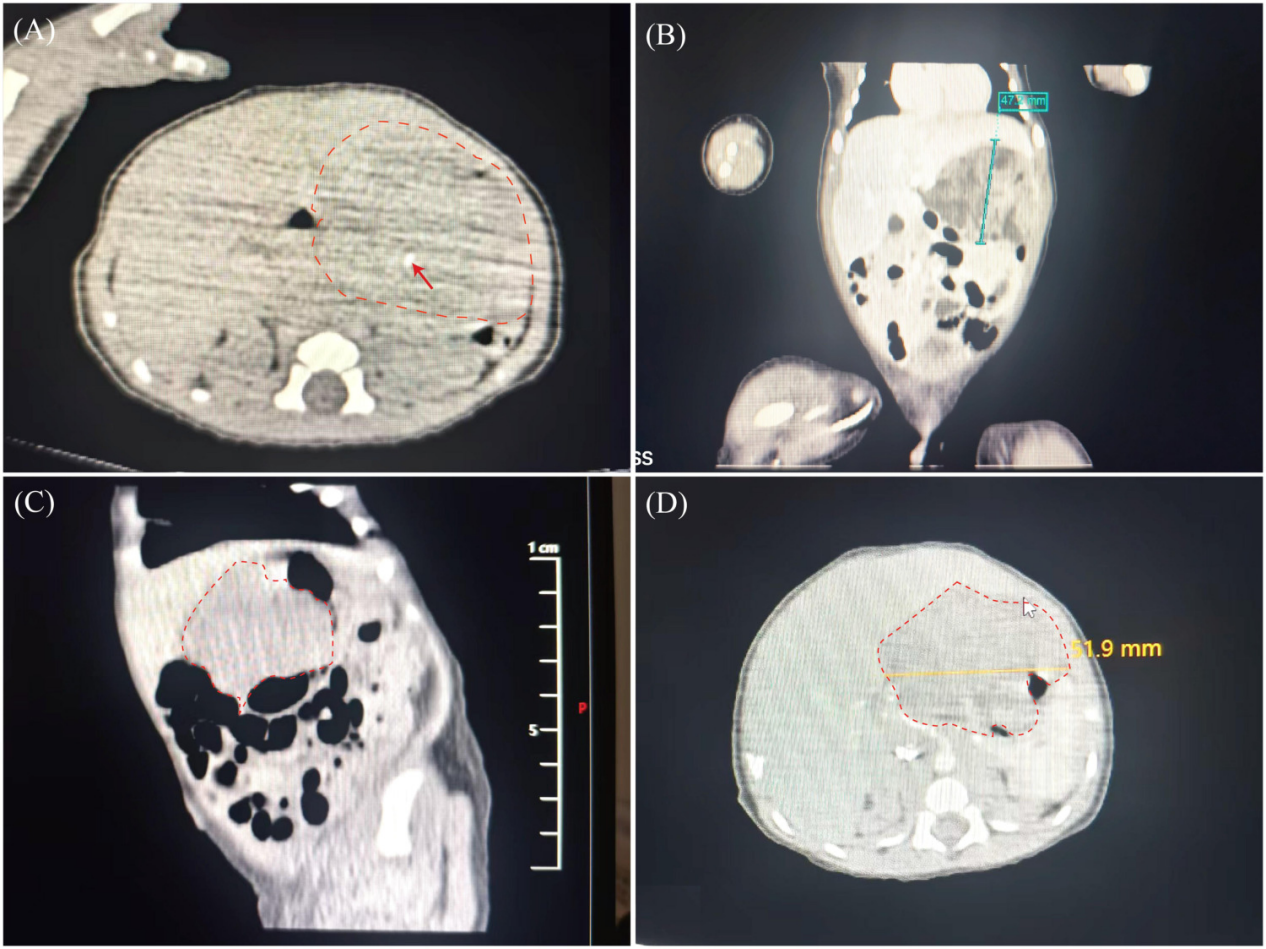
Preoperative abdominal computed tomography (CT) findings of a neonatal gastric teratoma arising from the anterior gastric wall. **(A)** Unenhanced axial CT image reveals a heterogeneous mass in the hepatogastric space containing a 2.9 mm hyperdense calcific focus (indicated by the red arrow). **(B)** Coronal and **(C)** sagittal contrast-enhanced CT reconstructions show the close association between the mass and the anterior gastric wall. **(D)** Contrast-enhanced axial CT shows the tumor measuring 51.9 mm in maximum diameter.

Non-contrast abdominal computed tomography (CT) performed on the first day of life revealed a large, heterogeneous, slightly hypodense lesion in the hepatogastric space, containing a 2.9 mm hyperdense calcific focus with poorly defined margins (Figure 1A). Contrast-enhanced CT demonstrated an irregular, heterogeneous mass measuring 51.9 mm × 47 mm × 39 mm, with internal calcific and fatty components. The solid portion of the lesion showed progressive moderate enhancement, and the mass was intimately associated with the anterior gastric wall (Figures 1B–D). Abdominal ultrasonography of the liver, gallbladder, pancreas, spleen, and kidneys revealed no additional abnormalities. Echocardiography demonstrated a patent foramen ovale with a left-to-right shunt measuring 1.2 mm in width on color Doppler. This finding was considered benign and did not require intervention during the current admission. Cardiac chamber dimensions, great vessel anatomy, and valvular function were within normal limits, with no other intracardiac anomalies identified. The presence of calcifications and fatty components within the mass was considered characteristic of a teratoma and served as a key imaging clue for the initial differential diagnosis.

### 2.3 Laboratory findings

Initial laboratory evaluation revealed tumor marker levels highly suggestive of a germ cell tumor. Serum AFP was markedly elevated at >60,500 ng/mL [normal reference range (NRR): 0–7 ng/mL], accompanied by a significant increase in NSE (63.8 ng/mL; NRR: 0–16.3 ng/mL) and lactate dehydrogenase (LDH) (575 U/L; NRR: 120–250 U/L). Carcinoembryonic antigen and carbohydrate antigen 19-9 were within normal reference ranges. Liver function tests showed mild transaminitis (aspartate aminotransferase: 55 U/L; NRR: 15–40 U/L; gamma-glutamyl transferase: 99 U/L; NRR: 10–60 U/L), mild hyperbilirubinemia (total bilirubin: 108.7 μmol/L; indirect bilirubin: 104.5 μmol/L), which was considered within the range of physiological jaundice in this neonate, and mild hypoalbuminemia (albumin: 35.0 g/L; NRR: 40–55 g/L) with a corresponding decrease in total protein (56.2 g/L; NRR: 65–85 g/L). A complete blood count revealed anemia (red blood cell count: 3.38 × 10^12^/L; NRR: 6–7 × 10^12^/L; hemoglobin: 119 g/L; NRR: 170–200 g/L). Inflammatory markers were notable for a significant elevation in interleukin-6 (IL-6) (15.73 pg/mL; NRR: 0–7 pg/mL) and a mild elevation in procalcitonin (0.123 ng/mL; NRR: <0.05 ng/mL), while high-sensitivity C-reactive protein (hs-CRP) was only mildly elevated (4.19 mg/L; NRR: 0–3 mg/L), with standard CRP within normal limits.

Postoperatively, a robust inflammatory response was observed. On day two, hs-CRP peaked at 37.7 mg/L and IL-6 at 24.87 pg/mL, accompanied by worsening anemia (hemoglobin: 104 g/L; red blood cell count: 3.21 × 10^12^/L) and mild thrombocytosis (345 × 10^9^/L). These markers showed a declining trend by postoperative day four, with hs-CRP decreasing to 13.9 mg/L, IL-6 to 13.63 pg/mL, and procalcitonin to 0.062 ng/mL.

### 2.4 Treatment and clinical course

Following comprehensive preoperative evaluation, the infant underwent surgical exploration on day four of life under general anesthesia. The operation proceeded as follows.

**Figure 2:**
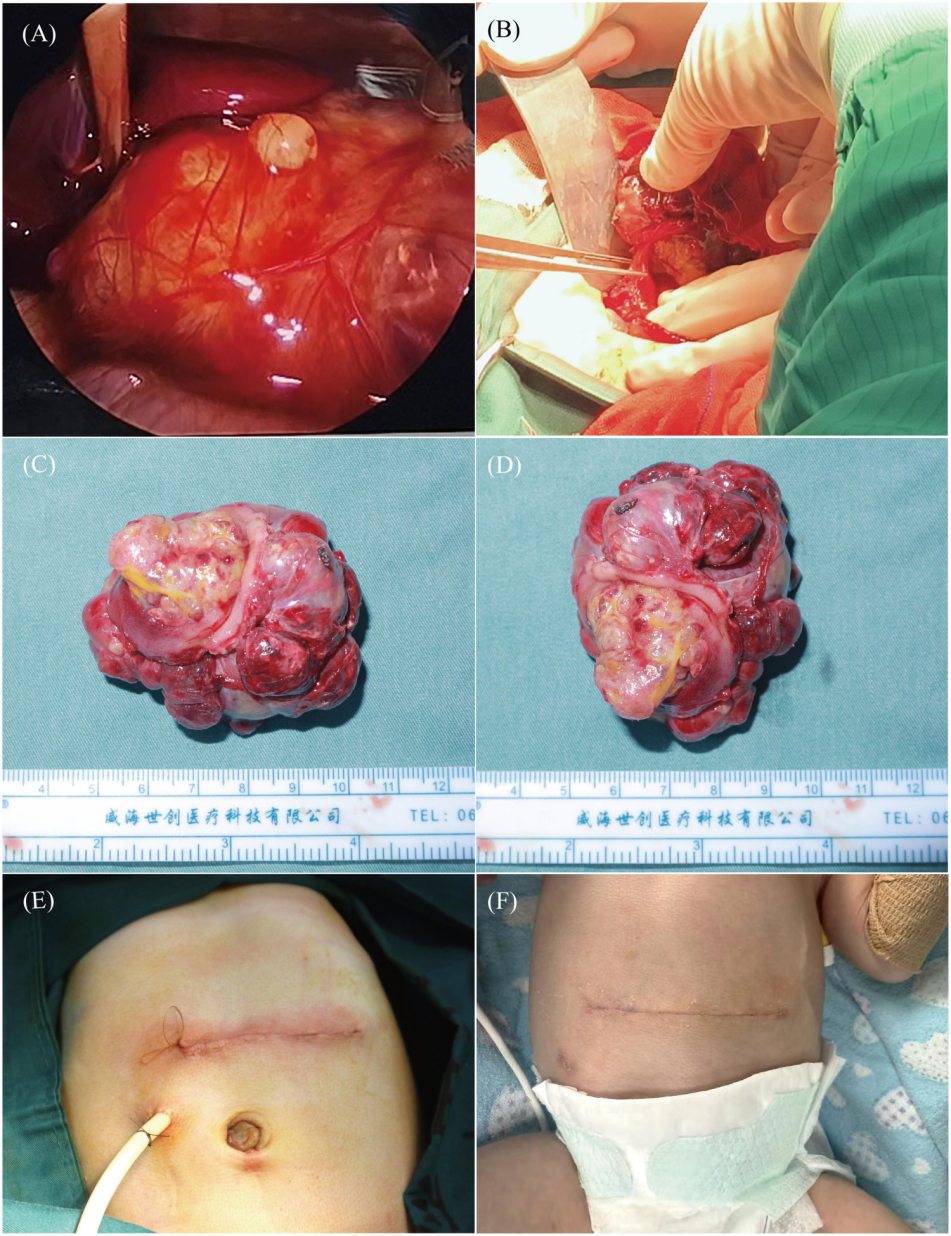
Intraoperative, postoperative and gross specimen findings of gastric teratoma resection. **(A)** Laparoscopic exploration reveals a tumor arising from the anterior gastric wall. **(B)** Resection of the anterior gastric wall tumor; transmural involvement was observed. **(C,D)** Gross appearance of the resected specimen. **(E)** Abdominal closure with a drain in place. **(F)** Postoperative appearance at one week.

Initial laparoscopic assessment via an infraumbilical incision revealed a 5.5 cm × 5 cm × 3.5 cm tumor occupying the hepatogastric space, intimately associated with the lesser curvature of the stomach. A small amount of hemorrhagic ascites was present, and the tumor was partially encased by hyperemic and edematous omentum (Figure 2A). Intraoperative exploration clearly confirmed that the tumor exhibited a transmural growth pattern involving the anterior gastric wall. Systematic exploration of the remaining abdominal cavity, including the colon and small intestine, revealed no evidence of additional malformations, metastatic deposits, or tumor infiltration. The infraumbilical incision was subsequently closed, and the procedure was converted to an open laparotomy to facilitate definitive resection.

The right and left gastric arteries were sequentially ligated to devascularize the tumor. A full-thickness circumferential gastrotomy was made in the anterior gastric wall, approximately 1.5 cm from the palpable tumor margin, and the mass was completely excised along its borders (Figure 2B). The distal resection margin was situated approximately 1 cm proximal to the pylorus. The gross specimen measured 5.5 cm × 4.5 cm × 3.5 cm (Figures 2C,D). The gastrotomy was closed in two layers using running absorbable sutures in a longitudinal orientation to ensure a watertight closure and minimize the risk of postoperative leakage.

Before abdominal closure, the peritoneal cavity was irrigated with warm sterile distilled water for 15 min, ensuring complete immersion of all visceral organs and the surgical field. A nasogastric tube was placed intraoperatively for gastric decompression, which prevented gastric distension and avoided leakage of gastric contents through the gastrotomy suture line during irrigation and abdominal closure. This hypotonic irrigation was performed to induce osmotic lysis of any potential microscopic residual tumor cells (4). The distilled water was thoroughly aspirated, followed by three cycles of irrigation with warm normal saline. After confirming meticulous hemostasis and the absence of any retained foreign material, the abdominal wall was closed in layers (Figure 2E). A 14-French closed-suction drain was placed adjacent to the gastric repair and exteriorized through the right abdominal trocar site.

Intraoperative blood loss was approximately 5 mL. To address the pre-existing anemia, the patient received a transfusion of 40 mL of fresh frozen plasma and 0.3 units of packed red blood cells. The procedure was uneventful, with stable hemodynamics throughout and no intraoperative complications.

Postoperatively, the infant was managed in the neonatal intensive care unit with prophylactic antibiotics, parenteral nutrition, and albumin infusion as clinically indicated. On postoperative day two, a small amount of coffee-ground-like flocculent material was observed in the abdominal drain; coagulation parameters were checked and found to be within age-appropriate normal limits. Nevertheless, 48 mL of fresh frozen plasma was administered to optimize coagulation status and reduce exudative output. The drain was removed on postoperative day six due to negligible output and clinical stability. The nasogastric tube was removed on postoperative day one week after observation of decreased and clear gastric fluid output. By the two-week follow-up, the infant was alert, hemodynamically stable, and tolerating full oral feeds without difficulty. The upper midline abdominal incision was well-approximated, with no erythema, drainage, wound dehiscence, or signs of infection, consistent with primary intention healing (Figure 2F).

### 2.5 Pathological results

**Figure 3:**
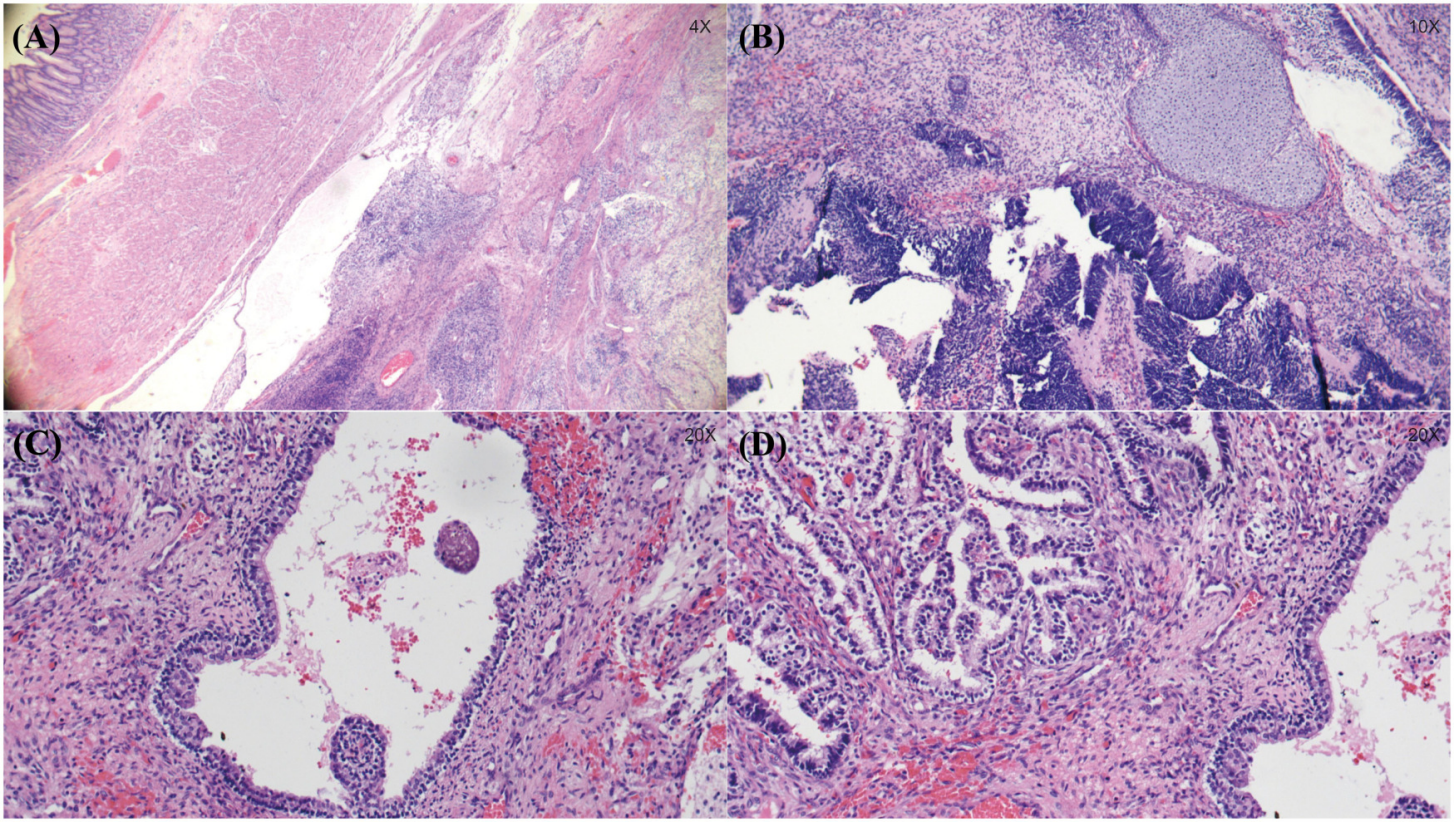
Histopathological features of grade III immature gastric teratoma (hematoxylin-eosin staining). **(A)** Low-power magnification (×4) shows the tumor arising from the gastric wall and extending beyond the muscularis propria into surrounding soft tissue; adjacent normal gastric mucosa is visible on the left. **(B)** Intermediate magnification (×10) demonstrates marked histologic heterogeneity, with mature hyaline cartilage of mesodermal origin adjacent to primitive neuroectodermal components. **(C)** Higher magnification (×20) reveals endoderm-derived glandular structures lined by pseudostratified ciliated columnar epithelium resembling bronchial mucosa, with surrounding stromal inflammation and hemorrhage. **(D)** Densely packed primitive glandular and tubular structures suggestive of gastroenteric or bronchiolar origin (×20). These findings are consistent with grade III immature teratoma.

Microscopic examination confirmed complete resection with negative margins (Figures 3A–D). The tumor contained tissues from all three germ layers, including adipose tissue, cartilage, and immature neuroepithelial tubules, consistent with a grade III immature teratoma.

## 3 Discussion

The diagnosis of GT hinges on cross-sectional imaging—predominantly CT, supplemented by x-ray—and serum tumor marker detection. Cross-sectional imaging reliably demonstrates the pathognomonic triad of calcifications, fat, and heterogeneous solid components, while serum tumor markers, particularly AFP, NSE, and LDH, serve as important auxiliary diagnostic indicators for germ cell tumors when analyzed comprehensively with clinical and imaging findings (5).

The present case exhibited all these classic features. Contrast-enhanced CT revealed a 5-cm hepatogastric mass containing calcifications and adipose tissue, which was closely associated with the anterior gastric wall on imaging; the tumor's transmural growth pattern was clearly verified during subsequent surgical exploration. Serum AFP exceeded 60,500 ng/mL, and NSE was markedly elevated at 63.8 ng/mL.

AFP is physiologically elevated in the neonatal period; its markedly high level in this case, when combined with clinical and imaging findings, served as a valuable auxiliary indicator for suspecting a germ cell tumor but not as definitive evidence of a malignant germ cell tumor. NSE is not a specific tumor marker for germ cell tumors, and its elevation was only considered an abnormal laboratory finding in the comprehensive diagnosis of teratoma, along with other markers, imaging, and intraoperative findings.

Considering the physiological elevation of AFP in neonates, the marked increase in AFP combined with elevated LDH served as an important auxiliary basis for the preliminary diagnosis of a germ cell tumor. As a non-specific marker, the elevated NSE was regarded as an abnormal laboratory finding and incorporated into the comprehensive diagnostic evidence alongside other indicators. These imaging and laboratory findings mirror those described by Zhang et al. (6), who reported a GT in an adult with similar radiologic characteristics, though their patient's tumor was confirmed to be a mature cystic teratoma on histology. In our case, pathologic examination subsequently confirmed the diagnosis of an immature gastric teratoma, consistent with previously reported features of this entity (3, 7).

On microscopic evaluation, the tumor was classified as a grade III immature teratoma, defined by the presence of abundant immature neuroepithelial elements exceeding four low-power fields per slide (8). The prognostic significance of histologic grading in GT remains unclear; unlike ovarian or testicular germ cell tumors, histologic grade does not reliably predict malignant potential or clinical outcome in GT (8). What matters prognostically—and what was achieved in this case—is complete, margin-negative resection without intraoperative spillage. Adjunctive measures, such as the intraperitoneal distilled water irrigation performed here to induce osmotic lysis of any residual microscopic disease, represent a prudent oncologic technique borrowed from adult gastrointestinal cancer surgery (4). Currently, there is no established indication for adjuvant therapy in neonates with completely resected, margin-negative immature gastric teratoma, as neonates are highly sensitive to the toxicities of chemotherapy and radiotherapy, and the potential risks outweigh the benefits. Despite complete margin-negative resection, the grade III histology and prominent immature neuroepithelial elements in our case justify prolonged, structured surveillance.

Laparoscopic exploration enables clear, magnified visualization of the tumor's location, size, and adjacent tissue involvement, facilitating a precise surgical resection plan and avoiding unnecessary injury to normal organs; it also allows accurate intraoperative assessment and excludes distant metastasis or other concurrent abdominal anomalies. This technique was valuable in our case for initial assessment before conversion to open laparotomy.

Long-term outcomes after complete resection of GT are generally excellent, and recurrence is unusual. However, given the immature histology in this case, structured surveillance is warranted. We follow the protocol proposed by Selvarajan et al. (9), with AFP measurements and cross-sectional imaging every three months for the first year, every six months for years two and three, and annually thereafter through seven years. The rare but sobering report of late recurrence 20 years after resection (10) underscores the need for prolonged vigilance; extending follow-up into adulthood may be reasonable for select patients.

Neonatal immature GTs are considered malignant or potentially malignant and carry a risk of recurrence. Complete surgical resection is the mainstay of treatment, and long-term regular follow-up is required.

Beyond its clinical features, this case illustrates the strengths of an integrated healthcare system. Prenatal detection at 37 weeks and 6 days of gestation triggered a coordinated response: postnatal transfer within 24 h, a complete diagnostic workup, and definitive surgical resection by day four of life. This rapid trajectory—from prenatal identification to postnatal intervention—exemplifies the paradigm increasingly advocated for rare neonatal tumors (11, 12). Prenatal ultrasound, long established for detecting structural anomalies, is now recognized as a critical tool for the early diagnosis of fetal neoplasms, enabling planned delivery at tertiary centers and prompt postnatal management (11, 12). Our experience reinforces this message and provides a template for the multidisciplinary care of similar cases.

## Data Availability

The original contributions presented in the study are included in the article/Supplementary Material, further inquiries can be directed to the corresponding author.
